# Prognosis of people with focal epilepsy treated with anti-seizure medications (ASMs): a narrative review of current evidence and future directions

**DOI:** 10.1136/bmjno-2025-001243

**Published:** 2025-12-16

**Authors:** Sarah Barnard, Emma Foster, Patrick Kwan, Jacqueline A French, Terence J O’Brien

**Affiliations:** 1 Monash University, Melbourne, Victoria, Australia; 2 Alfred Health, Melbourne, Victoria, Australia; 3 New York University, New York, New York, USA

**Keywords:** EPILEPSY, ANTICONVULSANTS

## Abstract

Epilepsy is a chronic, neurological disorder that affects 65 million people worldwide. Two-thirds are estimated to become seizure free with anti-seizure medications (ASMs), while one-third will ultimately develop drug-resistant epilepsy (DRE). Although >30 ASMs are now available, treatment outcomes among all epilepsies do not appear to have improved. There is a paucity of prognostic evidence specific to focal epilepsies, despite focal epilepsy being one of the most common forms of epilepsy and an independent risk factor for developing DRE. Accurate prognostication has largely been limited by variable definitions of treatment response in the literature. Use of the current International League Against Epilepsy treatment response definitions in research studies and clinical practice may improve the quality of prognostic evidence, as well as help identify important phenotypes of treatment responsiveness. This is particularly important as pharmacological treatments for epilepsy continue to expand without clear evidence we are improving outcomes.

## Introduction

 Epilepsy affects over 65 million people worldwide with 5 million people newly diagnosed each year.^1^ Approximately 60% of people with epilepsy have focal seizures,^2^ with focal epilepsy of unknown aetiology being the most common type of epilepsy.^3^ Having epilepsy is associated with a 2–7 fold risk of premature mortality and major lifetime morbidity, including poorer educational and employment outcomes, increased psychiatric disturbance and lower quality of life.^4 5^ The worldwide direct and indirect health cost of epilepsy is estimated to be greater than US$119 billion^6^ per year. The prognosis of people with epilepsy suggests two thirds will ultimately become seizure free with treatment with anti-seizure medications (ASMs).^7^,^9^ Despite optimal treatment, one in three people living with epilepsy will experience ongoing, recurrent seizures and may be considered to have drug-resistant epilepsy (DRE). The proportion of people with uncontrolled seizures is even higher in areas where people do not have dependable access to healthcare providers or epilepsy treatment.^1^ Small improvements in the rates of seizure freedom can significantly improve health and economic outcomes associated with epilepsy. An Australian cost analysis found just a 10% increase in the number of people with epilepsy reaching seizure freedom is predicted to save US$729 million in healthcare costs, prevent over 1600 deaths and provide productivity savings of US$5.3 billion dollars over a working lifetime.^10^ This article will present a narrative review of relevant prognostication data currently available on focal epilepsy, discuss key considerations in interpreting prognostication studies, and propose directions for future research.

### Search strategy

The following search terms were entered into electronic databases (PubMed, Scopus, OVID): “focal epilepsy”, “treatment OR anti-seizure OR anti-epileptic OR ASM OR AED”, “drug-resistan-“, “outcomes OR seizure response OR seizure outcome OR remission, long term OR seizure free”. Any study published in a peer-reviewed scientific journal in English language from 1974 to date of review (22 April 2025) was considered for relevance. Cohort, observational, prospective, retrospective, clinical trial study designs and editorials were included for review.

Forty-one studies were included for relevance. The major findings were extracted and synthesised into a narrative review as follows.

### About Focal Epilepsy

**Figure 1:**
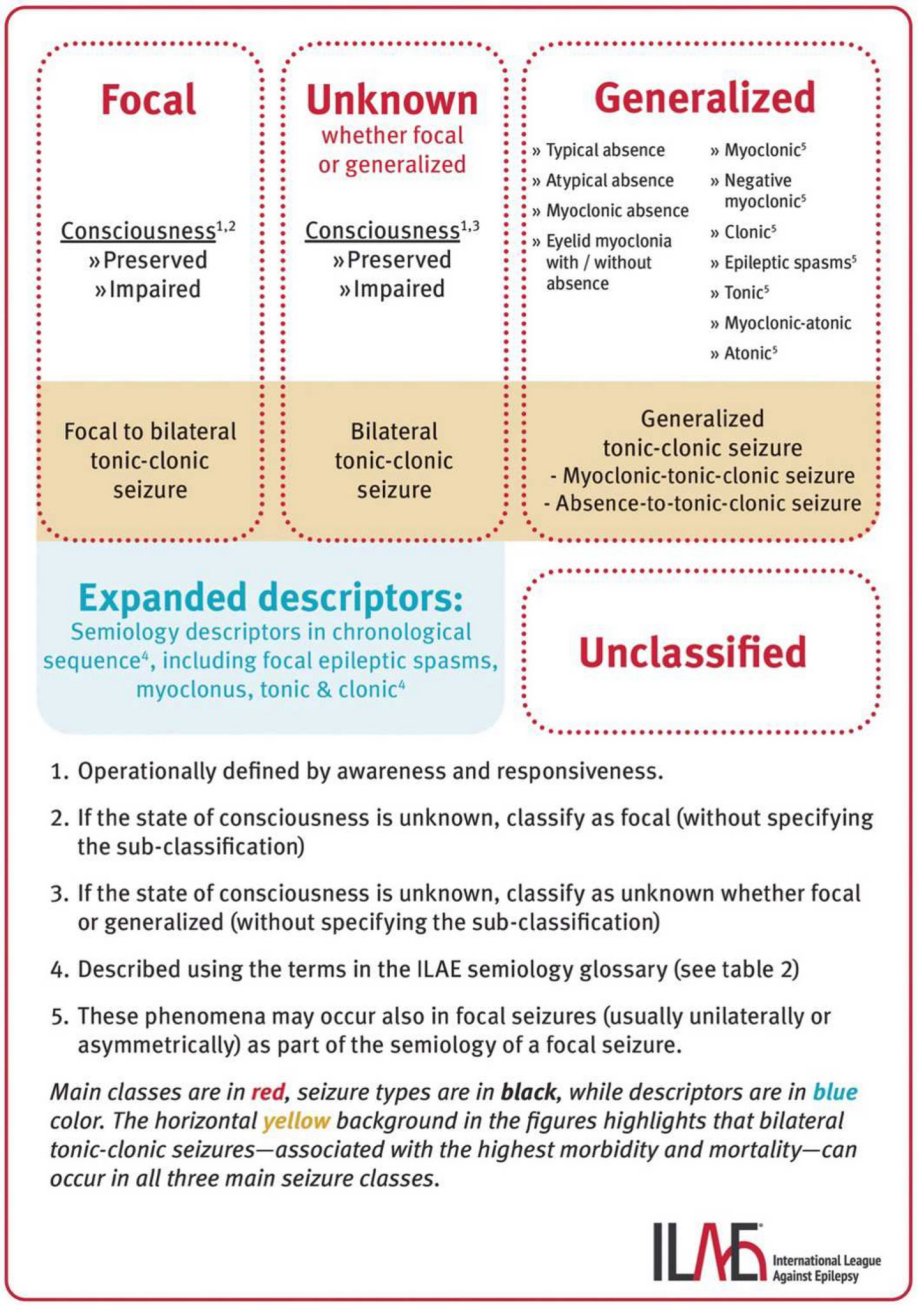
Current epilepsy and seizure classification system by the International League Against Epilepsy.^13^ Reproduced (unchanged) from Beniczky *et al*^13^ Updated classification of epileptic seizures: Position paper of the International League Against Epilepsy. *Epilepsia*. 2025; 00: 1–20. https://doi.org/10.1111/epi.18338. 2025 The Authors. Published by Wiley Periodicals on behalf of the International League Against Epilepsy. Licensed under a Creative Commons Attribution 4.0 International Licence (CC BY 4.0). The use of this figure does not imply endorsement by the authors or the publisher.

Epilepsy is broadly categorised into focal, generalised or combined focal and generalised epilepsies based on a patient’s seizure type(s)^11^,^13^ (figure 1). Focal epilepsy is the most common form of epilepsy in adults and children^3 14^ and describes seizures that originate in localised regions of the brain, usually limited to one hemisphere. These seizures may impair consciousness and/or have observable manifestations (focal with preserved consciousness±observable manifestations; focal with altered consciousness±observable manifestations (previously termed focal aware/simple partial or focal impaired aware/complex partial seizures)). They may also progress to involve bilateral hemispheres, resulting in a focal to bilateral tonic-clonic seizure (FBTCS) (previously termed ‘secondarily generalised tonic-clonic seizures’). The semiological classification of focal seizures is characterised by (1) the presence of observable signs (tonic, clonic, atonic, myoclonic, hyperkinetic or automotor movements) and (2) the level of consciousness (altered, preserved or unknown).^11^,^13^ The most common type of focal seizure is those with altered consciousness.^2^

The aetiology of most epilepsies, including focal epilepsies, is unknown (point prevalence 3.15 per 1000, vs 2.7 and 1.7 per 1000 for presumed structural and genetic causes, respectively).^1^ Where an aetiology is identified, it is most commonly related to structural or metabolic causes (2.7 per 1000 persons).^1^ The most common focal epilepsy syndrome is temporal lobe epilepsy (TLE).^15^ Seizures in TLE can present with an aura semiology, such as déjà vu, abdominal discomfort or epigastric rising sensation, anxiety/panic, or a feeling of impending doom (often seen preceding mesial temporal lobe seizures), or auditory sensations (more often seen in neocortical or lateral temporal seizures).^15 16^ Progression into altered consciousness, behavioural arrest, motor automatisms (e.g. lip smacking, chewing) or autonomic symptoms (e.g. tachycardia, flushing) can be seen in both mesial and neocortical temporal lobe seizures.^15 16^ While TLE has commonly been historically associated with hippocampal sclerosis, recently other pathological findings or non-lesional cases are becoming widely recognised.^17^ Focal epilepsies tend to follow a similar bimodal distribution of age seen among all epilepsies, with more people affected at younger and older ages. New-onset focal epilepsies in older persons are most often related to cerebrovascular disease, hypoxic brain injuries and cortical lesions such as tumours or vascular malformations.^14 18^ Common causes of new-onset focal epilepsies in children and neonates are self-limiting syndromes, or those related to structural, developmental or acquired aetiologies (e.g. perinatal brain injuries, trauma, vascular malformations, perinatal encephalopathies).^14 19^ Epilepsy of unknown cause tends to affect adolescents, young adults and middle-aged persons and often occurs in otherwise healthy individuals.^14^

ASMs are the first-line and mainstay treatment approach for managing focal and all epilepsies. Depending on the advisory body, the recommended first-line ASMs for focal epilepsy can include lamotrigine, carbamazepine, zonisamide or levetiracetam,^20 21^ though levetiracetam or zonisamide did not meet criteria for non-inferiority for efficacy and cost benefit in the 2021 SANAD trial.^22^ There is also emerging evidence for the use of cenobamate as add-on or monotherapy in focal epilepsy and brivaracetam as add-on therapy in drug-resistant focal epilepsy.^23 24^ While there may be higher rates of DRE in TLE,^15 25^ there is little evidence that there is a difference in the most effective ASMs between focal epilepsy syndromes.^22^ Adjunctive or polytherapy may be required in patients who fail to respond to serial monotherapy trials.

## Defining ‘treatment responsive’ & ‘treatment resistant’ epilepsy.

Evaluating response to medical treatment in epilepsy remains challenging. Epilepsy is a chronic, fluctuating condition that often requires lifelong ASM treatment to maintain seizure control. Seizure recurrence despite ASM treatment is common and many people will have seizures that relapse and remit over the course of their lifetime.^9^ Additionally, those who relapse tend to be indistinguishable in early treatment stages from those who have sustained seizure freedom.^9^ This makes it difficult to ascertain which patient groups need closer examination of their seizure patterns in the short and long term. There is also a high degree of variability in the severity of epilepsy both between and within epilepsy types. Additionally, the time required to assess treatment response can vary for those having frequent seizures at baseline (e.g. daily or weekly seizures) to those with less frequent seizures at baseline (e.g. yearly seizures). For some non-responders, total remission of seizures may not be achievable; however, a reduction in the frequency or severity of their seizures may be clinically meaningful. Relapses can also be related to reasons other than ASM efficacy, including poor drug adherence, lifestyle triggers (e.g. increased stress, alcohol intake or sleep deprivation), the natural course of a person’s epilepsy, the wrong diagnosis of an ongoing seizure type (e.g. functional seizures) or an inappropriate ASM for a patients’ seizure type (e.g. carbamazepine for generalised seizures). Adverse effects of ASMs are also common and likely contribute to apparent drug resistance due to early discontinuation and failures of treatments.^26 27^

### Treatment responsive

**Figure 2:**
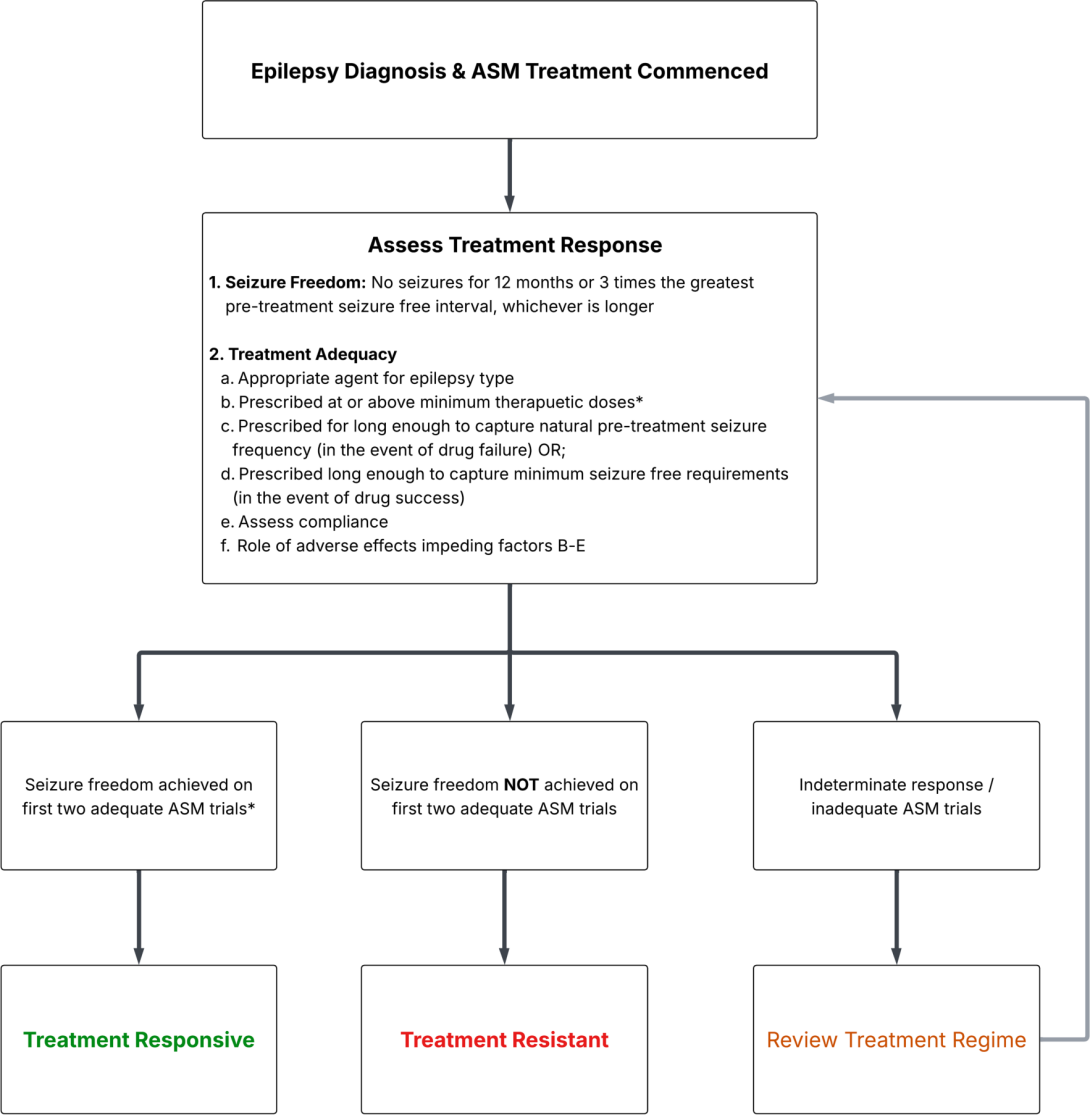
A proposed algorithmic pathway for assessing treatment response to anti-seizure medications (ASMs) in new-onset epilepsy, based on recommended definitions of seizure freedom, treatment response and treatment resistance by the International League Against Epilepsy. *Seizure freedom can be achieved on sub-therapuetic dosing as long as meeting ILAE minimum seizure free requirement.

Definitions of treatment response have varied within the literature but have most often been defined as ≥12 months without seizures on stable treatment, measured either at the start of treatment initiation or at the end of follow up. An International League Against Epilepsy (ILAE) 2010 consensus task force^28^ defined treatment responsive epilepsy as seizure freedom achieved on the first two adequate ASM trials (figure 2). In this context, ‘seizure freedom’ requires having no seizures for 12 months, or three times an individual’s greatest pretreatment interseizure interval (figure 2). An adequate ASM trial is one where an appropriate ASM for that epilepsy type was prescribed at or above minimum therapeutic doses, though a minimum dose for each ASM is not explicitly stated by the ILAE guidelines.

The ILAE’s minimum duration of seizure freedom is based on the ‘rule of three’, a statistical framework by Hanley and Lippman-Hand, which suggests that if a seizure has not occurred within three times the longest period of seizure freedom prior to treatment, there is a 95% likelihood that this is not by chance.^29^ This helps distinguish true treatment response from that of a natural inter-seizure interval fluctuation, which is particularly helpful for those who have infrequent seizures prior to treatment. Other than occurring following treatment, the ILAE definitions also do not stipulate when a seizure-free period can occur. This ensures all types of seizure freedom can be captured, including those with relapsing/remitting patterns and early or delayed seizure freedom. It also minimises the impact of variable drug titration schedules in early treatment stages and helps to capture delayed seizure freedom that may have occurred in people who may have drug resistance already established (i.e. seizure free after the failure of several ASMs).

The ILAE’s requirement for a person to respond to the first two ASMs to be considered a ‘responder’ is based on consistent evidence that the chance of achieving seizure freedom significantly decreases from the third ASM trialed onwards (though the odds are not zero).^7^,^9^ The addition that an ASM trial must be considered ‘adequate’ to count towards this cut-off may help ensure non-responsiveness to an ASM is not related to non-efficacy factors, such as the agent being prescribed or taken at sub-therapeutic doses or being an inappropriate choice for that person’s epilepsy type. This also implies the agent would need to be taken for long enough to assess success (seizure freedom) or failure (ongoing seizures).

### Treatment resistant

Treatment non-responsiveness has largely been reported in the literature in terms of an individual drug or intervention (treatment/drug failure), treatment resistance (the propensity to fail several drugs/interventions), or the persistence of seizures. As highlighted in a review by Perucca *et al*,^30^ treatment resistance is not synonymous with treatment failure, nor the persistence of seizures. This is because treatment failure (ie, ASM discontinuation) can occur for several reasons that are not related to seizure control. This includes inadequate medication adherence, including due to intolerable adverse effects, costs, drug availability or insurance/funding restrictions. Persistent seizures are also not synonymous with treatment resistance because seizures can persist in patients who are undertreated or non-adherent with medications. Undertreatment may occur due to subtherapeutic dosing (either through slow up-titrations to minimise adverse effects, or patient preference), too few ASM trials (ie, not switching to another agent after failing the first, usually due to access issues, patient preference, or practitioner preference to reach maximum dosing before switching), or due to insufficient time on a single agent to assess seizure response. With several ASMs available, there are many options for patients if they do not tolerate or fail prior ASMs. However, in practice, there is usually a limited amount of time a patient is willing to have ongoing seizures while trying multiple agents. For this reason, many patients will appropriately go down a treatment-resistant epilepsy evaluation and management pathway, including consideration of epilepsy surgery, after failing several ASMs irrespective of the reasons for failure.

The current definition of treatment resistance proposed by the ILAE consensus task force^28^ is the failure of two adequate ASM trials (figure 2). Failure of an ASM trial occurs with ongoing seizures on adequate dosing of an appropriate ASM for that epilepsy type. Discontinuation due to adverse effects or other non-efficacy reasons prior to recurrent seizures occurring is not considered treatment failure with respect to drug resistance, though it is noted that drug discontinuations are likely to occur for more than one reason (i.e. both adverse effects and inadequate efficacy). As described in the definition of treatment responsiveness, it can also be implied that if the ASM has not yet been failed, it would need to be maintained until failure occurs or the minimum seizure-free period has passed to be considered an adequate trial. This definition also implies that a patient can achieve seizure freedom after treatment resistance has been established (i.e. seizure freedom was achieved on third or more adequate ASM trial).

Few studies have employed the current ILAE definitions in a focal epilepsy population alone. A recent study on drug-resistant focal epilepsy^31^ patients found that when using the ILAE criteria, one in five cases of drug resistance was considered ‘pseudo-drug resistant’. This was attributed to discordance between an expert panel and the study investigators on what qualified as treatment failure and therefore drug resistance by ILAE criteria. There is also evidence that the addition of an extended seizure-free period beyond 12 months in those with infrequent pre-treatment seizures is not likely to alter results of previously published works.^7 32 33^ A recent meta-analysis reported that there is often insufficient data reported in prognostication studies to employ the ILAE criteria for treatment resistance.^32^ In one review, it was suggested that even longer than three times the longest pretreatment interseizure interval may be needed to capture true treatment response.^34^ Despite limited and mixed evidence for the utility for the application of the ILAE criteria, 12 months of seizure freedom remains associated with improved quality of life for most patients and may still provide a meaningful measure of treatment response where full outcomes are unattainable.^28^

## What are the odds a patient with epilepsy (of any type) will reach seizure remission with ASM treatment?

Based on a series of studies since the early 2000s looking at rates of seizure freedom in people with epilepsy, the prognosis of people who will ultimately become seizure free when treated with ASMs is estimated to be approximately two-thirds, while one third will go on to be treatment resistant (i.e. unable to have their seizures completely controlled with ASMs). One of the first seminal studies reporting this data was a large, single centre study by Kwan and Brodie.^8^ The authors prospectively assessed rates of seizure freedom of 470 people with newly diagnosed and treated epilepsy between the ages of 9 and 93 over a 13-year period in the 1980–1990s in Glasgow, Scotland. Defined by the absence or presence of seizure(s) in the 12 months or longer prior to last follow-up on stable treatment, they found 63% of patients were seizure free at follow-up and 37% were not. The authors also found the response to first-line ASM monotherapy was a good predictor of overall chance of treatment success. Approximately 47% of patients succeeded on their first agent, and the chance of achieving seizure freedom on successive ASM trials after failing the first was poor. The prescribing practices noted in their cohort at that time were a predominance of older generation ASMs (e.g. carbamazepine, valproate, phenytoin) and ASMs that are now established but were at the time newer generation agents (e.g. lamotrigine, gabapentin, oxcarbazepine).

A subsequent publication from the Glasgow cohort by Brodie *et al*^9^ characterised patients’ seizure and treatment outcomes using an extended cohort of 1098 newly treated epilepsy patients. Patients were followed up for a median of 7.5 years. This study defined seizure outcomes into four mutually exclusive seizure patterns: (A) early seizure freedom of 12 months or longer that commenced immediately or within 6 months of treatment initiation and was sustained until end of follow-up (37% of participants); (B) delayed seizure freedom of 12 months or longer, commencing after 6 months from treatment initiation and sustained until end of follow-up (22% of participants); (C) fluctuating course of seizure freedom for 12 months of longer with relapse and/or return of seizure freedom (16% of participants) and (D) never seizure free for a minimum of 12 months or longer (25% of participants). Patients who had a fluctuating treatment response could have up to 5 periods of relapse within the 7.5-year study follow-up period. Those who immediately became seizure-free had the same odds of relapse as those with delayed response. Of those who never became seizure free, 57% had tried more than two ASMs. Most patients in this study responded to their first ASM (68%). Survival analyses showed the chance of seizure freedom with successive ASM trials was again poor, and this was most pronounced for those with localisation-related epilepsies (focal, known aetiologies). Overall, the improvement in rates of seizure freedom between the cohorts was only 4% (64% to 68%), despite the introduction of 12 new ASMs in the 12-year period between publications. A further publication from this cohort by Chen *et al*^7^ showed that in an extended cohort of 1795 patients recruited between 1982 and 2012, 63.7% were seizure free in the 12 months preceding end of follow-up, but only 50% of patients achieved 12 months of seizure freedom on their first ASM. These outcomes were again only marginal, if at all, improved from previous, despite the predominance of newer generation agents. The likelihood of achieving 12 months of seizure freedom on the second or third ASM if the first agent failed remained poor (11.6% and 4.4% of those who tried a second or third agent, respectively). Failure of the first ASM (defined as discontinuation due to poor efficacy or adverse effects) was associated with 1.73 times increased odds of failing trials of subsequent ASMs. More recently, a systematic review and meta-analysis across 103 studies published between 1970 and 2020^35^ reported rates of treatment resistance to medical therapies in all epilepsies to be similar to the Glasgow cohorts at 20%–32%. However, the authors note there was moderately high variability on the reported rates of treatment resistance between studies, suggesting heterogeneity in study designs, definitions and population selection. The authors found that the greatest variance was related to age selection, definitions of ‘treatment resistance’ and duration of follow-up.

**Figure 3:**
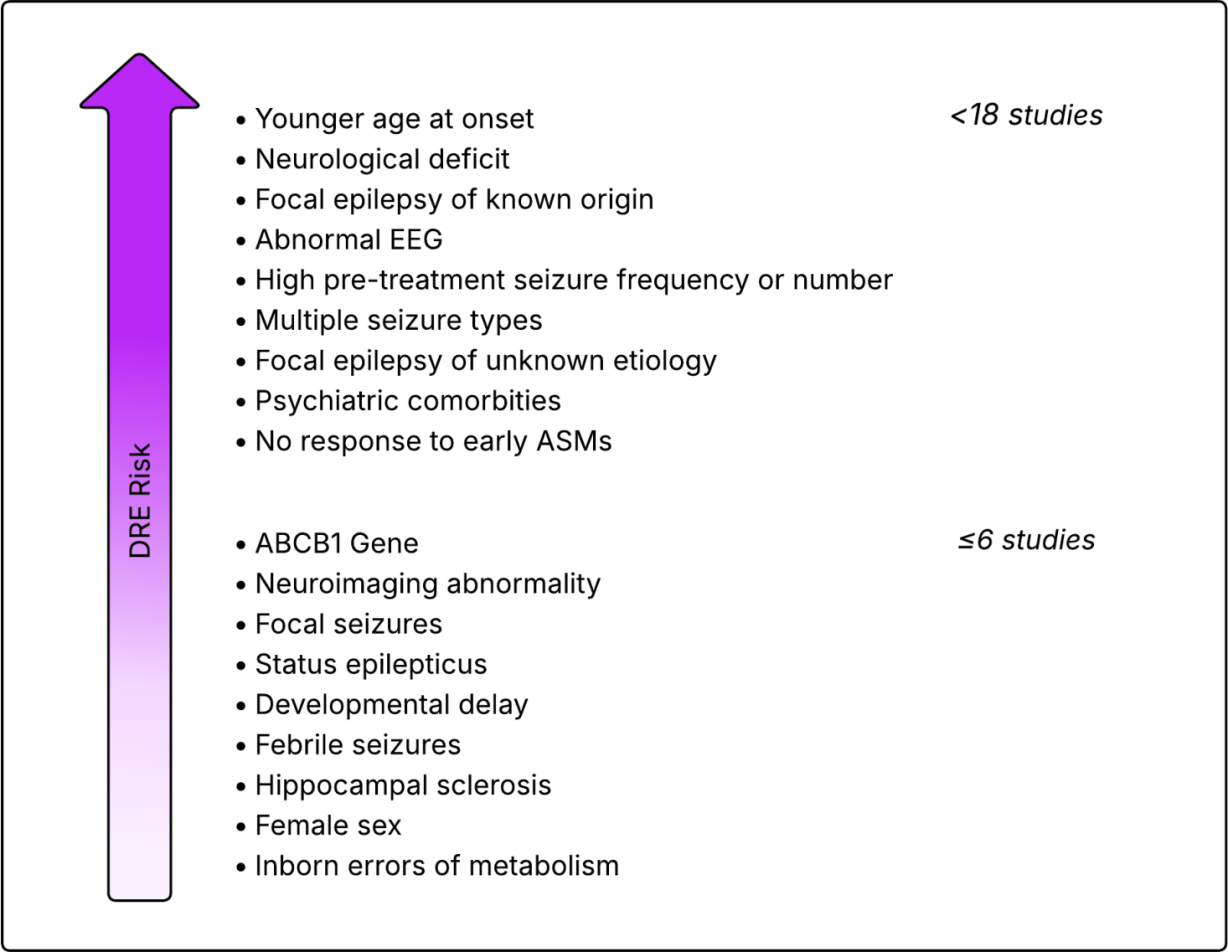
Risk factors for drug-resistant epilepsy reported in studies among all epilepsies in order of the number of studies that reported the factor as being associated with DRE, as identified in a systematic review and meta-analysis by Sultana *et al*.^35^ Figure adapted from Perucca *et al*.^30^ ASM, anti-seizure medications; DRE, drug-resistant epilepsy; EEG, electroencephalogram.

Within the literature, the most commonly reported risk factors for treatment resistance in all epilepsies (in order of frequency of studies who found an association) are younger age of onset, neurological deficits, symptomatic epilepsy, abnormal EEG, high baseline seizure number, presence of focal seizures, multiple seizure types, cryptogenic epilepsy (focal, unknown aetiology but presumed symptomatic) and psychiatric comorbidities^4 30 31^ (noting both symptomatic and cryptogenic epilepsies are no longer recommended terms of use by the ILAE) (figure 3). Psychiatric comorbidities in epilepsy are common, though they differ in prevalence among epilepsies^36^ and are associated with increased reporting of ASM adverse effects.^27^ As patients reach older ages, medication tolerance also decreases, which can increase the rate of drug failure.^37 38^ Having a higher number of pretreatment seizures is associated with an increased risk of poorer outcomes in several studies.^7^,^9^ This may reflect a worse epilepsy phenotype at onset that then precedes into treatment resistance. This hypothesis is supported by evidence from an Italian trial^39^ that showed treatment after a single seizure (versus two or more seizures) did not reduce the risk of developing future treatment resistance; however, a large proportion of this cohort also had indeterminate outcomes. Additionally, most studies reporting this association have done so by reporting patients’ pretreatment seizure number, rather than frequency. A discrete number (such as <5 or >5 pretreatment) does not account for the duration over which these seizures occurred and is therefore likely only a partial representation for disease burden. This distinction may be particularly important for patients with focal epilepsy who face significant delays to diagnosis, as focal non-motor seizures are often only recognised retrospectively to be seizures following a first presentation with a focal to bilateral tonic-clonic seizure.^40^ We also know treatment failure, by definition, can be assessed more quickly than treatment success. This definition bias will be more pronounced for those who have frequent seizures at baseline than someone who has less frequent seizures, as well as in studies with shorter follow-up periods.

## What are the odds a patient with focal epilepsy will reach seizure remission with ASM treatment?

Despite focal epilepsy and the presence of focal seizures being independent risk factors for treatment resistance among all epilepsies, there is a paucity of studies assessing the prognosis of people with focal epilepsy as a group. The SANAD II Trial^22^ indirectly reported on the treatment outcomes of people with newly diagnosed focal epilepsy as part of a randomised control study to evaluate non-inferiority of levetiracetam and zonisamide against lamotrigine as first-line agents in focal epilepsy. This trial recruited 990 patients with newly treated focal epilepsy across the UK and performed a post hoc time-to-event analysis for 12-month seizure remission (defined as days from drug randomisation to the date at which a period of 12 months had elapsed without the patient having any seizures) and treatment failure (defined as days from randomisation to the date a decision to withdraw the randomised drug, or to add a new ASM because of inadequate seizure control or unacceptable adverse reactions). Although overall rates of seizure remission and treatment failure were not explicitly reported, survival curve analyses suggest that by around 5 years, 70%–80% of patients will reach seizure remission on one of these agents (levetiracetam, zonisamide or lamotrigine, with levetiracetam having the lowest rate) and about 40% will reach remission in the first year of treatment (i.e. seizure free immediately from treatment onset). By 5 years, 30%–70% will have failed one ASM (with levetiracetam and zonisamide more likely to be failed due to adverse effects).

A follow-up analysis of this cohort with an extended group of 1721 newly diagnosed focal epilepsy patients in 2012^41^ assessed risk factors for treatment failure. In this study, the authors similarly defined treatment failure as poor seizure control or unacceptable adverse effects leading to the discontinuation of that drug or the addition of a second agent, as measured by time to treatment failure. Risk factors for earlier treatment failure were female sex, presence of prior ASM trials, younger age (<10 years), greater number of pretreatment seizures (4–11 versus 2 or less), abnormal EEG, the absence of a focal to bilateral tonic-clonic seizure type and poorly localised epilepsy. Treatment failure due to poor efficacy alone was increased in those with previous ASM trials, younger age and greater pretreatment seizures. Being female, restarting epilepsy treatment following a period of remission, older age and absence of a focal to bilateral (‘secondarily generalised’) tonic-clonic seizure type was associated with treatment failure due to adverse effects alone. Supportive of the findings from the Glasgow cohorts, a recent multicentre, observational, prospective study^31^ on 1053 drug resistant focal epilepsy also found the odds of focal epilepsy patients achieving seizure freedom with successive ASM trials (after already failing two ASMs) was poor. Defined as the absence of seizures for 12 months or three times an individual’s greatest pre-treatment interval, the authors found only 11.8% of participants went on to achieve seizure freedom on their third ASM, and this dropped to 2.6% following six or more ASM failures.

## Where to from here?

The current landscape of prognostication in epilepsy suggests we have not clearly improved outcomes for people with epilepsy despite 30 ASMs currently available on the market. As focal epilepsies are a large and phenotypically distinct group of disorders, more prognostication data is needed on treatment outcomes. Evaluating our progress in successfully treating focal and all epilepsies with ASMs has been limited by variable definitions of treatment response in epilepsy research. It has also suffered from a lack of data on the complexities of treatment factors that affect the efficacy and tolerability of ASMs, and therefore, treatment success.

Quantifying and reporting nuanced treatment outcomes in focal epilepsy by use of the new ILAE criteria may reveal the presence of more diverse epilepsy phenotypes within focal epilepsy (and other epilepsies). This may include patients who will respond to any medical therapy (‘treatment sensitive’), only specific medical therapy (i.e. ‘treatment intolerant’ or ‘treatment selective’), or few medical therapies (‘treatment resistant’). Identifying these phenotypes at or around the time of diagnosis may help optimise early treatment choices, so patients can reach a determinable treatment outcome sooner. This may look like switching to other drug classes early after first drug failure (due to efficacy, adverse effects or both) in those identified as likely treatment intolerant or selective, or early surgical referral for those likely to be treatment resistant. This approach will likely improve the chances a patient will be moved onto a more efficacious treatment earlier, reducing the significant mortality and morbidity associated with uncontrolled seizures. The identification of these groups will also better inform clinicians of a patient’s risk of seizures in different treatment periods, including the implications this has on counselling patients who drive, work, attend school or are engaging in activities deemed high risk with epilepsy (e.g. swimming, sleeping alone). This may be particularly pronounced for those with new-onset focal epilepsy in young or mid-life who regularly participate in these activities.

## Challenges and future directions

As the current ILAE treatment outcomes definitions become employed in more research studies, researchers face new challenges of addressing the inherent subjectivity that comes with their greater nuance. Studies may need to focus on optimising inter-rater reliability when operationalising the ILAE definitions, as well as ensuring study duration follow-up is sufficient to capture the high variability of seizure burden within epilepsy syndromes and types. The employment and evaluation of these criteria in emerging studies will have implications for future drug development, including how treatment response will be measured in clinical trials.
